# Diagnosis and Treatment of Laryngeal Extranodal Natural Killer/T-cell Lymphoma

**DOI:** 10.7759/cureus.96497

**Published:** 2025-11-10

**Authors:** Gustavo Quispe-Villegas, Yinno Custodio, Rolig Aliaga

**Affiliations:** 1 Medicine, Cayetano Heredia University, Lima, PER; 2 Oncohematology, Arzobispo Loayza National Hospital, Lima, PER

**Keywords:** extranodal nk-t-cell, laryngeal neoplasms, larynx, lymphoma, non-hodgkin lymphoma

## Abstract

Extranodal natural killer (NK)/T-cell lymphoma (ENKL) is an aggressive subtype of non-Hodgkin lymphoma (NHL). Its presentation in the larynx is extremely rare and may mimic tuberculosis and squamous cell carcinoma. Laryngeal ENKL is a rare form of NHL, often diagnosed at an advanced stage with a poor prognosis despite chemoradiotherapy. We report the case of a 26-year-old female patient with an eight-month history of fever, cough, hoarseness, and dysphagia. Contrast-enhanced CT revealed a mass protruding from the left posterior pharyngeal wall. Immunohistochemistry confirmed the diagnosis of laryngeal ENKL. The patient received CHOP chemotherapy (cyclophosphamide 700 mg, doxorubicin 50 mg, vincristine 2 mg, dexamethasone 20 mg), which was later changed to DeVIC (dexamethasone 40 mg, etoposide 70 mg, ifosfamide 1,000 mg, carboplatin 200 mg) with local radiotherapy. After completing treatment, the patient was readmitted four months later with multiple episodes of fever, hemoptysis, and odynophagia. Due to the inability to place a nasogastric tube, a Witzel gastrostomy was performed. However, the patient died from non-oncological causes.

## Introduction

Extranodal natural killer (NK)/T-cell lymphoma (ENKL) is a rare and aggressive subtype of non-Hodgkin lymphoma (NHL), characterized by the presence of T and NK cells, an angiocentric growth pattern, and a strong association with the Epstein-Barr virus (EBV) [1]. It predominantly affects the upper aerodigestive tract and exhibits a male predilection with a 3:1 ratio, most commonly presenting in the fifth decade of life [2-4]. ENKL of the upper aerodigestive tract is often classified as the nasal type; it comprises up to 80% of all ENKL cases and 2.4% of all NHL cases in Peru [5-7]. This category includes ENKL located in the nasal cavity, paranasal sinuses, pharynx, and larynx [1,2,8,9]. In contrast, 10-15% of all ENKL cases are classified as the extranasal type, which primarily affects the gastrointestinal tract, skin, salivary glands, and testes [1,8,9].

At the laryngeal level, squamous cell carcinoma is the most common cause of laryngeal cancer [10]. In contrast, nonepithelial cancers account for only 1% of cases and consist mainly of plasmacytomas, diffuse large B-cell lymphomas, and mucosa-associated lymphoid tissue lymphomas [11-13]. The prevalence of B-cell lymphomas over T-cell lymphomas is 6:1, making laryngeal ENKL only 1% of all ENKL cases [6,10]. Thus, laryngeal ENKL is exceedingly rare and can mimic the presentation of laryngeal squamous cell carcinoma [13]. Here, we report a rare case of laryngeal ENKL in a female patient with an unfavorable outcome despite therapeutic intervention and initial response.

## Case presentation

A 26-year-old female presented with an eight-month history of sore throat, dry cough, odynophagia, dysphagia, hoarseness of voice, anorexia, and 26 lb of weight loss. She had no night sweats or hemoptysis. Additionally, there was no relevant epidemiological contact, and no relatives had similar symptoms. The patient had COVID-19 in 2020 without requiring oxygen support. She had no family history of hypertension, type 2 diabetes, Hashimoto's thyroiditis, or NHL. Six months before admission, she underwent upper endoscopy due to persistent odynophagia and dysphagia, which revealed purulent material and diffuse ulceration on the posterior oropharyngeal wall, with preservation of the esophageal mucosa. The patient did not undergo treatment due to diagnostic uncertainty, as a biopsy was not conducted.

On admission, the patient presented with worsening symptoms and a fever of 40°C. Marked intolerance to both liquids and solids was noted. On physical examination, the patient was in fair condition with normal vital signs. Oropharyngeal assessment revealed congestion without secretions or plaques. No palpable masses or lymphadenopathy were detected. No abnormalities were noted in the skin, abdomen, pulmonary, cardiovascular, or neurological systems. Specifically, there were no signs of ulcerations, rashes, petechiae, hemorrhagic diathesis, hepatosplenomegaly, pallor, jaundice, focal deficits, paresthesia, or paresis. A laryngeal endoscopy was performed using a 70° rigid endoscope, revealing purulent lesions on the left posterior wall, originating from a supraglottic mass that obstructed visualization of both true vocal folds. This led to the presumptive diagnosis of laryngeal tuberculosis, given the endemic setting.

**Figure 1:**
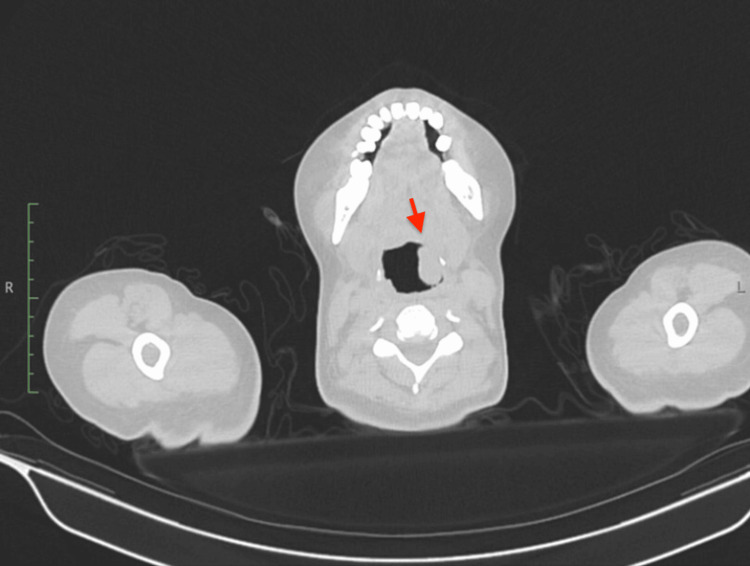
Contrast-enhanced CT of the head and neck, axial plane A concentric, asymmetric 18 x 49 mm mass arising from the left oropharyngeal mucosal wall resulted in 60% airway stenosis (red arrow).

Complete blood count results were unremarkable. Serological testing for HIV-1 and HIV-2 antibodies was negative, and nontreponemal tests were nonreactive. Coagulation studies, as well as folate and vitamin B12 levels, were within normal limits. None of the six sputum acid-fast bacilli smears yielded a positive result. Further immunological evaluation revealed normal complement C4 and antistreptolysin antibody levels. Contrast-enhanced CT of the head and neck identified a concentric and asymmetric mass measuring 18 x 49 mm in the left mucosal oropharyngeal wall, with extension into the supraglottic and glottic regions of the larynx. The lesion involved both the true and false vocal cords, leading to 60% airway stenosis. No lymphadenopathy was observed (Figure 1).

**Figure 2:**
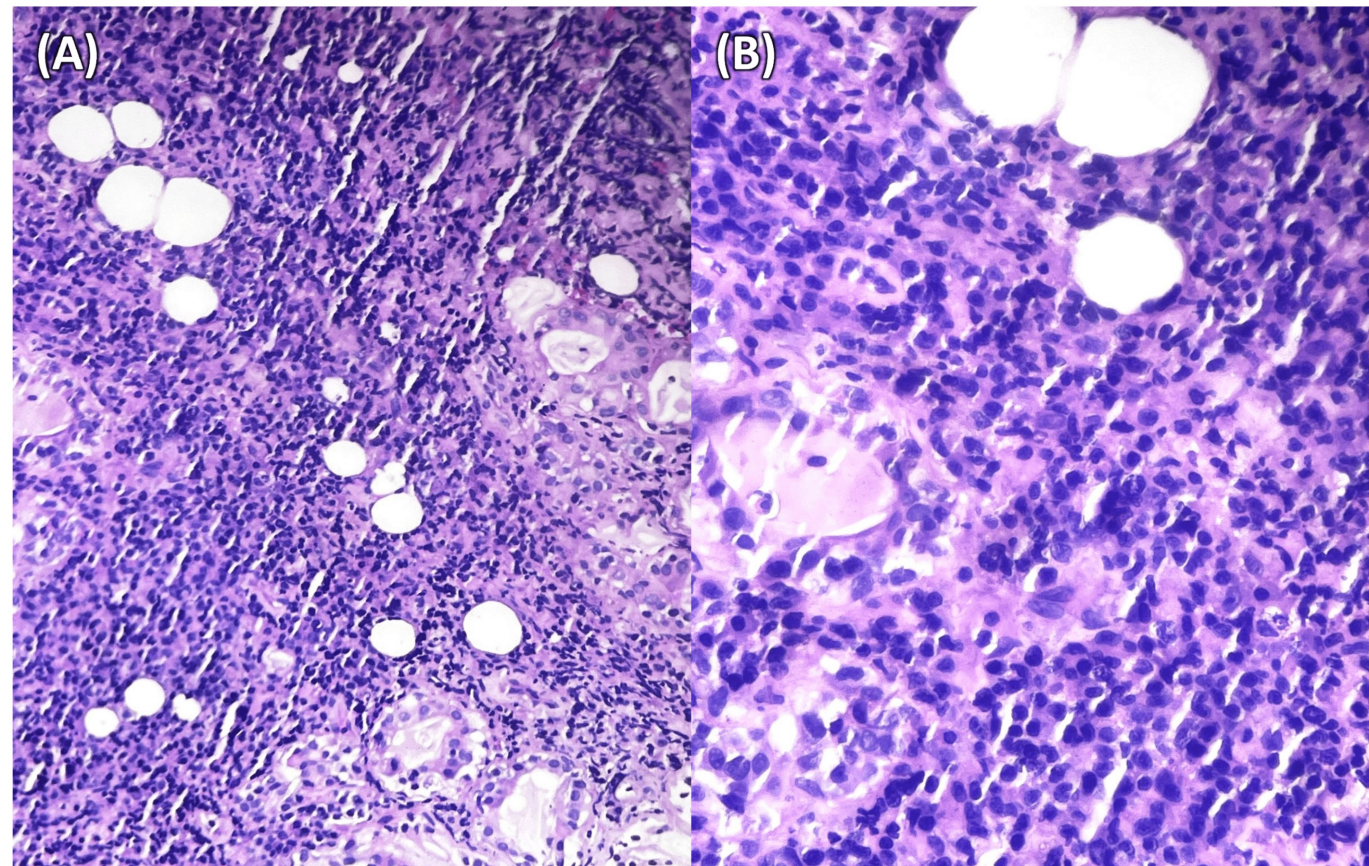
Histopathological findings H&E staining revealed a lymphomatous infiltrate with monomorphic small round cells. (A) H&E 100x magnification; (B) H&E 400x magnification H&E: Hematoxylin and eosin

A subsequent excisional biopsy was performed via laryngoscopy. Intraoperatively, a friable tumor with a fibrinous appearance was excised, which was primarily located on the left side, without complications. Histopathological examination with hematoxylin and eosin (H&E) staining revealed extensive hemorrhagic areas. A dense, monomorphic infiltrate of small, round lymphoid cells with high nuclear-to-cytoplasmic ratios and inconspicuous nucleoli was observed. The infiltrate exhibited an angiocentric pattern, leading to vascular destruction and areas of coagulative necrosis. No granulomas or lymphoid follicles were present (Figure 2).

**Figure 3:**
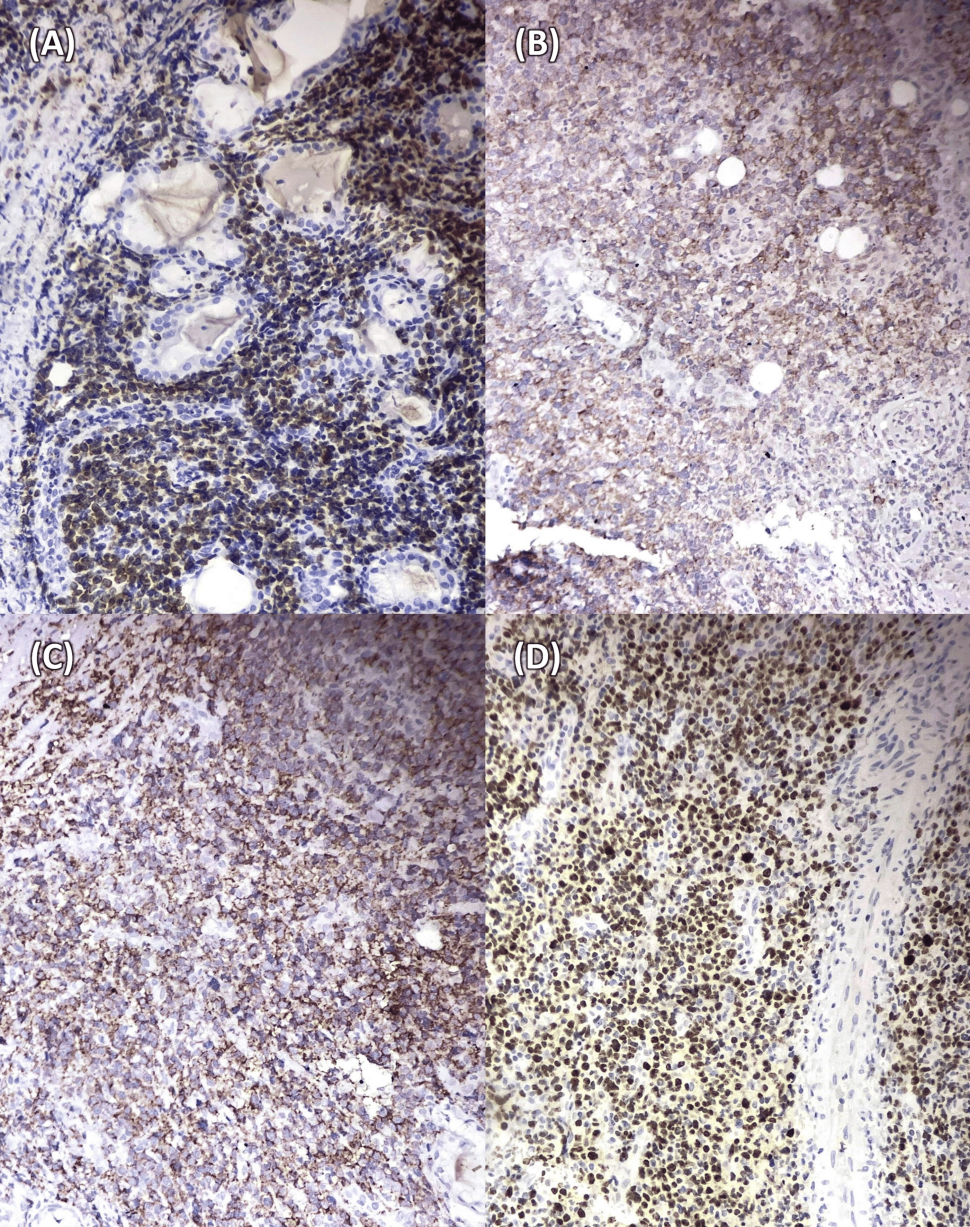
Immunohistochemical panel An immunohistochemical panel showed the same lymphocytic population expressing (A) CD3, (B) CD30, (C) CD56, and (D) a Ki67 proliferation index of 40%. 100x magnification.

Immunohistochemistry identified a lymphocytic population that was CD3 (+), CD20 (-), and pan-cytokeratin (-), with a Ki-67 proliferation index of 40%. These findings supported the initial classification of a T-cell lymphoproliferative process. Serial contrast-enhanced CT scans of the chest, abdomen, and pelvis showed no evidence of lymphadenopathy or metastatic lesions. A second immunohistochemistry panel with the initial biopsy sample revealed a population of CD56 (+), CD3 (+), CD30 (+), CD4 (-), CD8 (-), CD20 (-), EBV (-), and a Ki-67 proliferation index of 40% (Figure 3). Based on these findings, the case was further classified as ENKL, nasal type.

Based on the initial immunohistochemistry findings and for symptomatic relief, a nasogastric tube was placed without incident, and the patient was initiated on an emergency CHOP chemotherapy regimen (cyclophosphamide 700 mg, doxorubicin 50 mg, vincristine 2 mg, dexamethasone 20 mg) for a suspected T-cell lymphoma. Although initial improvement was observed in dysphonia and dysphagia, subsequent evaluation with the second immunohistochemistry panel (which confirmed ENKL) necessitated a switch to DeVIC chemotherapy (dexamethasone 40 mg, etoposide 70 mg, ifosfamide 1,000 mg, carboplatin 200 mg) for three cycles. The patient was discharged, and outpatient follow-up was scheduled for chemoradiotherapy. Following the first cycle, the patient was hospitalized in the ICU of another institution for 20 days due to acute respiratory failure secondary to severe pneumonia. After discharge, the patient received a second cycle of DeVIC plus filgrastim. Local radiotherapy was administered in 28 fractions with 6 MV photon beams at a dose of 50.4 Gy, with adequate tolerance. The patient completed her third cycle of DeVIC without any reported incidents. Therefore, a follow-up nasolaryngoscopy was subsequently performed. Granulomatous fibrosis was found with improvement of the lesions at the level of the posterior wall of the pharynx, and the vocal folds exhibited poor mobility with a limited range of motion toward the midline. The nasogastric tube was replaced without incident.

The patient was readmitted four months later, presenting with fever episodes ranging from 38°C to 40°C that started one week prior. Upon re-admission, she had a productive cough with hemoptysis and odynophagia. After removal of the nasogastric tube, a notable presence of pus was observed. Upper endoscopy revealed multiple confluent ulcerations with irregular borders, covered in fibrin and friable tissue, involving the upper esophageal sphincter and proximal esophagus. Due to the inability to place a nasogastric tube, the patient received parenteral nutrition and subsequently underwent a Witzel gastrostomy. However, the patient ultimately died from post-surgical bleeding.

## Discussion

Compared to the 2-12% prevalence of NHLs, ENKL accounts for up to 3-10% of NHLs in Asia and South America [3,4,13]. The latter is attributed to ethnic predispositions in populations from Asian and Latin American countries, as ENKL represents 3-10% of all malignant tumors in East Asia and is the most prevalent lymphoma in individuals of Amerindian descent, as observed in our patient [1,14]. In contrast, ENKL is rare in Western regions, constituting less than 1% of all lymphomas in these countries [12,15]. According to the International Peripheral T-cell Lymphoma Project, the incidence of ENKL is four times higher in Asian countries than in Western countries (22% vs. 5%) [8].

The distinction between nasal and extranasal ENKL is crucial because the latter corresponds to the sites where metastases of the nasal type typically spread, resulting in significantly inferior survival rates [2,5]. According to the International Peripheral T-cell Lymphoma Project, extranasal ENKL has a reported median survival of 0.36 years, whereas nasal ENKL has a median survival of 1.6 years [9]. Laryngeal ENKL is classified as a nasal type, in contrast with other studies reporting it as part of the extranasal type [1-3,8,9,11,14]. However, despite the distinctive difference in survival rates, there is no variation in age, sex, ethnicity, or immunophenotype between the two ENKL types [6].

Immunohistochemistry is essential for confirming the presence of ENKL. This is demonstrated by the positivity of CD56, granzyme B, perforin, and cytoplasmic CD3 or epsilon antigen [3,4,7,8]. The latter must be distinguished from membrane CD3, which is negative in ENKL but can be detected in conjunction with cytoplasmic CD3 when using polyclonal antibodies, as observed in this case [2]. Additionally, CD30 positivity is present in 20-50% of cases, which is associated with a better prognosis, despite the fatal outcome in our patient [16]. On the other hand, ENKL is negative for CD4, CD8, and CD20, exhibiting a double negative T phenotype shared by peripheral T-cell lymphomas, not otherwise specified [3,13]. Although these tumors may also express CD56, the median age of presentation at 60 years, distinct clinical presentation, frequent nodal involvement, and lack of CD3 epsilon expression suggest that this diagnosis was unlikely in our patient [17].

ENKL exhibits a robust association with the EBV, which is present in episomal form during latency stage II, similar to Hodgkin lymphoma, with the expression of latency antigens such as Epstein-Barr Nuclear Antigen 1 and Late Membrane Protein 1 [2,5,8]. EBV antigens may be detected by immunohistochemistry, but the primary method for confirming viral infection at the tumor level is through in situ hybridization of EBV-encoded nuclear RNA (EBER) [8]. This method not only facilitates the detection of viral genomic material but also correlates directly with tumor burden, providing prognostic value due to the proportional release of EBER during tumoral apoptosis [5,8]. The presence of EBV is universal in ENKL, as the viral episome is clonally integrated, suggesting that the infectious event precedes lymphomagenesis [2,6,12]. Conversely, the absence of EBV via EBER in situ hybridization rules out ENKL as a diagnosis [1].

In this case, EBER in situ hybridization was not performed due to the unavailability of the test at our hospital, and the presence or absence of EBV DNA was neither confirmed nor ruled out. Moreover, following chemotherapy, false-negative results may occur due to a reduced tumor burden. As observed in other NHLs, EBER-negative results can arise from previously EBER-positive tumors that have recurred [18]. For this reason, based on the clinical presentation, pathology findings, and initial response to the DeVIC regimen and radiotherapy, the diagnosis of laryngeal ENKL was favored.

## Conclusions

Laryngeal ENKL is a rare subtype of NHL, often diagnosed at an advanced stage and associated with a poor prognosis. It is frequently misdiagnosed as more common conditions, such as laryngeal tuberculosis, further delaying appropriate treatment. Diagnosis primarily relies on histopathological examination, immunohistochemical profiling, and EBER in situ hybridization, though resource limitations may hinder definitive testing. Despite early detection and prompt intervention with multimodal strategies combining chemotherapy and radiotherapy, patients remain at high risk of relapse, complications, and poor survival outcomes.
